# An Energy-Efficient Two-Stage Cooperative Routing Scheme in Wireless Multi-Hop Networks

**DOI:** 10.3390/s19051002

**Published:** 2019-02-26

**Authors:** Jianming Cheng, Yating Gao, Ningbo Zhang, Hongwen Yang

**Affiliations:** School of Information and Communication Engineering, Beijing University of Posts and Telecommunications, Beijing 100876, China; chengjm@bupt.edu.cn (J.C.); YTGao@bupt.edu.cn (Y.G.); nbzhang@bupt.edu.cn (N.Z.)

**Keywords:** wireless multi-hop network, two-stage cooperative transmission, cooperative routing, network lifetime, energy efficiency

## Abstract

Cooperative routing is one of the most widely used technologies for improving the energy efficiency and energy balance of wireless multi-hop networks. However, the end-to-end energy cost and network lifetime are greatly restricted if the cooperative transmission model is not designed properly. The main aim of this paper is to explore a two-stage cooperative routing scheme to further improve the energy efficiency and prolong the network lifetime. A two-stage cooperative (TSC) transmission model is firstly designed in which the core helper is introduced to determine the helper set for cooperation. Then, the two-stage link cost is formulated where *x*, the weight of residual energy, is introduced to be adjusted for different design goals. By selecting the optimal helper set, the two-stage link cost of each link can be optimized. Finally, based on the designed TSC transmission model and the optimized two-stage link cost, a distributed two-stage cooperative routing (TSCR) scheme is further proposed to minimize the end-to-end cooperative routing cost. Simulation results evaluate the effect of *x* on the different performance metrics. When *x* equals 0, TSCR can achieve the shortest end-to-end transmission delay and highest energy efficiency, while a larger *x* can achieve a longer network lifetime. Furthermore, simulation results also show that the proposed TSCR scheme can effectively improve both the energy efficiency and network lifetime compared with the existing schemes.

## 1. Introduction

In recent years, the development of wireless multiple-hop technology provides a promising direction for the wireless sensor network or ad hoc network [1] where each node is equipped with a wireless transceiver to exchange data with other neighboring nodes and route packets via neighboring nodes to destinations that are not within direct communications if necessary [2,3]. However, the majority of nodes in the wireless sensor network or ad hoc network are usually power-constraint and deployed in unattended environment where people have difficulty in replacing or recharging the depleted devices. For this reason, it is especially important to save the energy cost and keep the network energy-balanced [4] for the end-to-end packet transmission schemes. To address these problems, the cooperative communication [5,6,7] was introduced to increase the energy efficiency of end-to-end transmission by allowing several single-antenna nodes to collaborate with each other and forward the packet to the destination. Furthermore, cooperative routing is a cross-layer routing scheme by combining cooperative communication in the physical layer and routing technology [8] in the network layer to select a cooperative route and allocate power for nodes in the route. According to the different design goals, the previous works can be mainly classified into two categories: the first one is to maximize the end-to-end performance of wireless multi-hop transmission, the other is designed to maximize the network lifetime.

Several attempts [9,10,11,12,13,14,15] have been made to maximize the end-to-end performance of wireless multi-hop transmission, such as minimizing total energy routing (MTE) [9], cooperation along the minimum energy non-cooperative path (CAN) [10], progressive cooperative routing (PC) [10], cooperative cluster-based routing (CwR) [11], cooperative shortest path algorithm (CSP) [12], relay selection-based cooperative routing (CC-OPT) [13], power efficient location-based cooperative routing (PELCR) [14] and minimum-energy cooperative routing (MECR) [15]. MTE [9] aimed at finding a minimum total energy non-cooperative route and can be achieved by a standard shortest path algorithm [16] such as Dijkstra and Bellman-Ford. In CAN-*l* [10], the last *l* nodes along the above non-cooperative route cooperatively transmit the packet to the next-hop node. Similar to CAN-*l*, PC-*l* [10] combined the last *l* nodes into a single node and then updated the shortest path from the combined node to the destination node. CwR [11] introduced the ‘recruiting-and-transmitting’ phase based on the non-cooperative path, and the cooperative model was similar to the multiple-input-single-output (MISO) case. The above schemes were designed based on the minimum energy non-cooperative path. In fact, the effect of cooperation on the performance should be considered in searching the optimal end-to-end route. Based on CAN-*l*, CSP [12] was designed to find an optimal cooperative shortest path considering the gains of *l*-to-1 cooperative transmission. However, the channel model of this scheme only considered the effect of distance and ignored the fading of wireless environment. In CC-OPT [13] and PELCR [14], a relay node of each cooperative link was elaborately selected from the nodes located in the middle area between the transmitter and the receiver. However, the number of cooperative nodes should be adaptive to the actual neighbors instead of a predefined number. MECR [15] designed the cooperative link cost subject to a constraint on link reliability and proposed a probabilistic cooperative routing to find the minimum-energy route in the network, but it is executed in a centralized manner and has a high computational complexity.

A considerable amount of literature [17,18,19,20,21,22,23] has been published on maximizing the network lifetime. These studies suggest that the network lifetime reflects the energy balance of the whole network and is generally defined as the time when the energy of the first node becomes depleted. The flow augmentation (FA) [18] designed the link cost considering the residual energy of node which avoided the low-power node in the best route and prolonged the network lifetime. Referring to CAN-*l*, the flow augmentation cooperative routing (FACR) [19] further combined the cooperative *l*-to-1 communication case and residual energy of transmitters to formulate the cooperative link cost, but the selection of cooperative transmitters should be extended from the best route to their neighbors. The energy-balanced cooperative routing (EBCR) [20] assumed that each node can estimate its lifetime based on the expected energy consumption during each end-to-end transmission, and the transmitter of single-hop communication could greedily pick those nodes with larger remaining lifetime to be helpers of the current link, which would balance every helper’s remaining lifetime after the transmission. In fact, the estimation of expected energy cost of each node is not accurate for ignoring the shadowing fading of wireless channel. In [21], the maximization of network lifetime was investigated by designing a weighted sum-power minimization strategy, and the multihop transmission was modeled in a amplify-and-forward manner which will result in the problem of noise propagation and be unfavorable to designing the cooperative routing. The lifetime maximization cooperative routing with truncated automatic repeat request (LMCRTA) [22] predefined a possible relay for each link, and the link cost was designed considering the cooperative link and the retransmission. In the virtual multiple-input-multiple-output-based cooperative routing (VMIMOCR) [23], the virtual node and virtual link were defined respectively by taking the cooperation of two neighbors into consideration, and based on these definitions, the cooperative routing algorithm was designed to maximize the network lifetime. However, the number of cooperative nodes in LMCRTA and VMIMOCR schemes should not be fixed.

Motivated by these, this paper proposes an energy-efficient two-stage cooperative routing (TSCR) scheme to simultaneously improve the energy efficiency and prolong the network lifetime. In contrast with the existing schemes, the *importance and originality* of this study are that it firstly explores a two-stage cooperative (TSC) transmission model where the core helper is introduced to determine the helper set for cooperation. Then, the two-stage link cost is formulated where the effect of cooperative transmission is considered, and the weighting coefficient of residual energy, *x*, is introduced to accommodate the different design goals. By selecting the optimal helper set, the two-stage link cost of each link can be optimized. Finally, based on the optimized two-stage link cost, a distributed TSCR scheme is further designed to minimize the end-to-end route cost and establish the shortest path between source and destination, then the source packet is delivered to the destination via this path with the proposed TSC transmission model. The proposed TSCR scheme can adapt to the fading environment and meet different performance requirements. The benefits of TSCR protocol in network residual energy, network lifetime, average end-to-end transmission delay and energy efficiency are investigated in comparison with the existing schemes through simulation results.

The rest of the paper is organized as the following. In Section 2, we describe the system model as well as the existing schemes. Section 3 introduces our proposed TSCR scheme. The performance evaluation results of TSCR scheme are presented in Section 4. Finally, the paper is concluded in Section 5.

## 2. System Model and Existing Schemes

### 2.1. System Model

We consider a wireless network consisting of *N* nodes arbitrarily distributed in an area, where each node is equipped with a single omnidirectional antenna. These nodes can self-organize to form a multi-hop network. Define the whole network as a connected undirected graph $G\;=\;\left(V,P\right)$, in which V is the set of nodes and $\left|V\right|\;=\;N$ is the number of nodes. P denotes the set of all the bi-directional wireless communication links between pairs of nodes. In case a node i∈V transmits data *X* with the maximum power $P_{\max}$, and another node j∈V can successfully decode *X* without the aid of any other nodes, we say a link $P_{i,j}\in P$ exists. Assuming that *X* is encoded with an ideal forward error correction (FEC) code, then $P_{i,j}\in P$ implies that if node *i* transmits with maximum power, the received signal-to-noise ratio (SNR) at node *j* is at least $\gamma_{\min}$ which is dictated by the FEC code-rate.

**Figure 1:**
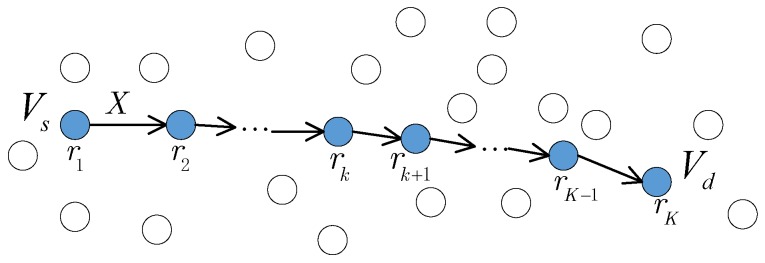
A route from source to destination.

Consider a pair of arbitrarily chosen source node $V_{s}\in V$ and destination node $V_{d}\in V$ as shown in Figure 1. The data of $V_{s}$ is delivered to $V_{d}$ via a route $r=\left\{r_{1},r_{2},\ldots ,r_{K}\right\}$ where $r_{1}=V_{s}$, $r_{K}=V_{d}$, $r_{2},\cdots ,r_{K-1}\in V$ are intermediate relay nodes such at $P_{k,k+1}\in P$. Each hop is assigned with a *link cost* $C_{k,k+1}$. The shortest path is given by

(1) $$\begin{array}{c} r^{*}=\underset{r\in \Omega \left(V_{s},V_{d}\right)}{\arg\min}\sum_{k=1}^{K}C_{r_{k},r_{k+1}}, \end{array}$$

where $\Omega \left(V_{s},V_{d}\right)$ is the set of all possible routes from $V_{s}$ to $V_{d}$. The difference of various routing schemes mainly lies in the different definition of link cost and different transmission model in each hop.

With the non-cooperative point-to-point transmission model, the signal received by node *j* from node *i* can be expressed as

(2) $$\begin{array}{c} y_{j}=h_{i,j}w_{i}X+z_{j}, \end{array}$$

where $z_{j}$ is complex Gaussian noise with zero mean and variance $\sigma^{2}$. *X* is the data symbol satisfying $E[|X{|}^{2}]=1$. $w_{i}$ is the power control coefficient such that $|\omega_{i}{|}^{2}=P_{i}\leq P_{\max}$. $h_{i,j}$ denotes the complex channel gain between node *i* and *j*. We assume that the channel between any two nodes is Rayleigh block fading, and $E[{\left|h_{i,j}\right|}^{2}]$ is inversely proportional to $d_{i,j}^{\alpha }$ [24], where $d_{i,j}$ denotes the distance between node *i* and *j*, α is the path loss exponent. The received SNR at node *j* is given by

(3) $$\begin{array}{c} \gamma_{j}=\frac{{\left|h_{i,j}w_{i}\right|}^{2}}{\sigma^{2}}=\frac{P_{i}{\left|h_{i,j}\right|}^{2}}{\sigma^{2}}. \end{array}$$

If node *i* can reach *j*, i.e., $P_{i,j}\in P$, then by definition there must be $\frac{P_{i}{\left|h_{i,j}\right|}^{2}}{\sigma^{2}}\geq \gamma_{\min}$. The *Neighbor Set* of node *i*, $N\left(i\right)$, consists of all reachable nodes of *i*:

(4) $$\begin{array}{c} N\left(i\right)=\left\{j\in V\left|\frac{P_{\max}{\left|h_{i,j}\right|}^{2}}{\sigma^{2}}\geq \gamma_{\min}\right.\right\}. \end{array}$$

Similar to [4,5], we assume that within the range of N(i),∀i∈V, channel state information (CSI) is available to node *i* and the signaling messages can be transmitted reliably with the negligible cost.

Due to the broadcast nature of wireless communication, it is possible that when node *i* transmit *X* with power $P_{i}\leq P_{\max}$, multiple nodes in N(i) can decode *X*. Hence, some of the hops in Figure 1 may benefit from the cooperative transmission of multiple nodes. The concept of cooperative transmission has already been discussed in multi-hop routing schemes [25,26] in the form of cooperative beamforming model.

Let $T=\left\{t_{1},t_{2},\cdots ,t_{l}\right\}$ be a subset of N(j) such that all nodes in T have already decoded *X* in the previous transmission. With the cooperative beamforming model, the signal received at *j* can be expressed as

(5) $$\begin{array}{c} y_{j}=\sum_{t\in T}h_{t,j}w_{t}X+z_{j}, \end{array}$$

where $\omega_{t}$ is beamforming coefficient satisfying $|w_{t}{|}^{2}=P_{i}\leq P_{\max}$. The received SNR is given by

(6) $$\begin{array}{c} \gamma_{j}=\frac{{\left|\sum_{t\in T}h_{t,j}w_{t}\right|}^{2}}{\sigma^{2}}. \end{array}$$

Similar to [10,19], we assume that each time there is only one active communication session which is the one from $V_{s}$ to $V_{d}$ as shown in Figure 1. In case there are multiple active flows, the cooperative beamforming should address the interference that may occur at some relay nodes, for example with multi-channel operation [27,28], interference aware beamforming [27,29], non-orthogonal multiple access (NOMA), local scheduling, full-duplex operation [30,31], etc.

We also assume that the network is quasi-stationary such that the topology of network and the channel coefficients do not change or change slowly so that the cost for topology discovery and CSI acquirement is negligible. In addition, we assume that the data volume of traffic data is much larger than the signalling message, hence the energy of nodes is mainly consumed in data delivery phase rather than the routing setup phase. We also assume that all nodes have the same initial energy $E_{\mathrm{ini}}$ and can adjust its transmit power $P_{i}$ continuously up to the maximum limit $P_{\max}$.

### 2.2. Existing Schemes

This subsection introduces serval existing routing schemes including MTE [9], PC-l [10] which focus on minimizing the end-to-end transmission energy consumption, and FA [18], FACR [19] which are designed to maximize the network lifetime. The main difference between these schemes lies in the definition of link cost $C_{i,j}$ and the transmission model in each hop.

**MTE transmission scheme** [9] defines $C_{i,j}^{\mathrm{MTE}}$ as the minimum transmission power cost to achieve a packet transmission between node *i* and *j*,

(7) $$\begin{array}{c} C_{i,j}^{\mathrm{MTE}}=\min\left\{\left.{\left|w_{i}\right|}^{2}\right|\gamma_{j}\geq \gamma_{\min}\right\}=\frac{\gamma_{\min}\sigma^{2}}{{\left|h_{i,j}\right|}^{2}}. \end{array}$$

**PC-l transmission scheme** [10] introduces the multipoint-to-point cooperative model as Equation (6). In this case, the link cost is designed as the minimum transmission power cost to form the cooperative link (T,j)

(8) $$\begin{array}{c} C_{T,j}^{\mathrm{PC}}=\min\left\{\left.\sum_{t\in T}{\left|w_{t}\right|}^{2}\right|\gamma_{j}\geq \gamma_{\min}\right\}=\frac{\gamma_{\min}\sigma^{2}}{\sum_{t\in T}{\left|h_{t,j}\right|}^{2}}, \end{array}$$

where the last equation comes from the Cauchy-Schwartz inequality.

The PC-l scheme starts from establishing a non-cooperative route $r^{\left(1\right)}\;=\;\left\{V_{s},r_{2}^{\left(1\right)},\cdots ,V_{d}\right\}$ with Equations (1) and (7). Then the source $V_{s}$ sends data to next node $r_{2}^{\left(1\right)}$ along the path and combines the first two nodes into a super node $T=\left\{V_{s},r_{2}^{\left(1\right)}\right\}$. Next, the link costs between the super node and other nodes are calculated with Equation (8), and the route is updated to $r^{\left(2\right)}=\left\{T,r_{2}^{\left(2\right)},\cdots ,V_{d}\right\}$. This process continues and at the $k^{\mathrm{th}}$ hop, the super node will contain all last min{l,k} nodes, i.e., $T\;=\;\left\{r_{2}^{\left(k-l\right)},r_{2}^{\left(k-l+1\right)},\;\cdots \;,r_{2}^{\left(k-1\right)}\right\}$ if k>l, or $T\;=\;\left\{V_{s},\;\cdots \;,r_{2}^{\left(k-1\right)}\right\}$. All nodes in T transmit the same data to $r_{2}^{\left(k\right)}$ with the cooperative beamforming model. It is obvious that MTE is a special case of PC-*l* with l=1.

**FA transmission scheme** [18] aims at improving the network lifetime and considers the normalized residual energy of transmitting node in calculating the link cost, i.e.,

(9) $$\begin{array}{c} C_{i,j}^{\mathrm{FA}}=\min\left\{\left.{\left|w_{i}\right|}^{2x_{1}}\cdot \frac{E_{\mathrm{ini}}^{x_{2}}}{E_{i}^{x_{3}}}\right|\gamma_{j}\geq \gamma_{\min}\right\}={\left(\frac{\gamma_{\min}\sigma^{2}}{{\left|h_{i,j}\right|}^{2}}\right)}^{x_{1}}\cdot \frac{E_{\mathrm{ini}}^{x_{2}}}{E_{i}^{x_{3}}}, \end{array}$$

where $E_{i}$ is the residual energy of node *i* at current time, $E_{\mathrm{ini}}$ is the initial energy of node *i*, and $\left(x_{1},x_{2},x_{3}\right)$ are parameters that govern the effect of normalized residual energy. It can be seen from Equations (7) and (9) that MTE is a special case of FA with $\left(x_{1},x_{2},x_{3}\right)=\left(1,0,0\right)$.

**FACR transmission scheme** [19] introduces cooperative transmission into FA to further improve the network lifetime. The procedure of FACR is exactly the same as PC-*l* except that the link cost is replaced by

(10) $$\begin{array}{c} C_{T,j}^{\mathrm{CR}}=\min\left\{\left.\sum_{t\in T}\frac{E_{\mathrm{ini}}}{E_{t}}{\left|w_{t}\right|}^{2}\right|\gamma_{j}\geq \gamma_{\min}\right\}=\frac{\gamma_{\min}\sigma^{2}}{\sum_{t\in T}\frac{E_{t}}{E_{\mathrm{ini}}}{\left|h_{t,j}\right|}^{2}}. \end{array}$$

It should be noted that in the cooperative routing schemes like PC-*l* and FACR, nodes in transmitter set T should have the CSI related to the receiver node to perform the cooperative beamforming. This implies that T is within the neighbor set of receiver.

PC-*l* and FACR can considerably improve the network performance compared with their non-cooperative version MTE and FA. However, these schemes have excluded the nodes that are not in the current non-cooperative path from participating the cooperative transmission. By no means we can say that the last *l* nodes in the non-cooperative route is the optimal transmission set for the $k^{\mathrm{th}}$ hop data transmission. In addition, PC-*l* and FACR need to re-solve Equation (1) at each hop, using Dijkstra and Bellman-Ford algorithm for example. This may introduce the excessive delay and signalling overhead. In the next section, we will propose a two-stage cooperative routing scheme to solve these problems.

## 3. Proposed Two-Stage Cooperative Routing

In this section, we firstly design a TSC transmission model and propose a two-stage link cost in which the core helper plays a significant role for each link. Then, the selection of core helper is detailed and a TSCR algorithm based on the optimized two-stage link cost is proposed to search the optimal cooperative shortest path. It is noted that the proposed TSCR is also designed based on the general routing model as Equation (1), in contrast with the existing schemes, the cooperative transmission model is redesigned and the link cost is reformulated to achieve a better cooperative routing.

### 3.1. Two-Stage Cooperative Transmission Model

Before the introduction of the proposed cooperative model, some definitions of the basic elements for the two-stage transmission are given as follows.

1. **Candidate core helpers**$U_{i,j}$: If any node *u* is reachable to node *i* and has better channel condition than the receiver node *j*, node *u* will become a candidate core helper. The set of *Candidate core helpers* $U_{i,j}$ can be expressed as
  (11) $$\begin{array}{c} U_{i,j}=\left\{u\in N\left(i\right)\left|\left|h_{i,u}\right|>\left|h_{i,j}\right|\right.\right\}. \end{array}$$
2. **Core helper**$u_{i,j}$: Suppose that node $u_{i,j}\in U_{i,j}$ is selected as the *Core helper node* for the hop i→j. When node *i* has a data to transmit to node *j*, the broadcast power of node *i* will depend on the channel condition between node *i* and $u_{i,j}$. For simplicity of notation, drop the index {i,j} in the following definition, in this case, the broadcast power $P_{T}$ can be calculated as
  (12) $$\begin{array}{c} P_{T}=\left\{\begin{array}{c} \frac{\gamma_{\min}\sigma^{2}}{{\left|h_{i,u}\right|}^{2}},\;u\in \left\{U_{i,j}/i\right\}\\ 0,\;u=i \end{array}\right., \end{array}$$
  where u=i stands for the situation where *i* will transmit data directly to *j* without any helper.
3. **Helper set $T\left(u\right)$**: If node *i* broadcasts the data with node *u* as the core helper, the *Helper set* $T\left(u\right)$ is a set of nodes which are reachable to both *i* and *j* and have better channel condition than the core helper *u*, i.e.,
  (13) $$\begin{array}{c} T\left(u\right)=\left\{t\in N\left(i\right)\cap N\left(j\right)\left|\left|h_{i,t}\right|\geq \left|h_{i,u}\right|\right.\right\}. \end{array}$$
  When u=i, $T\left(u\right)=\left\{i\right\}$.

Suppose that node *i* has a data *X* to transmit to node *j* (i,j∈V), in the proposed scheme, the data transmission can be divided into the following two stages:

- **Stage 1:** Node *i* determines the candidate core helpers $U_{i,j}$ and selects the core helper *u* from $U_{i,j}$. $T\left(u\right)$ depends on the selection of core helper *u*. Then, node *i* broadcasts the packet *X* to the helper set $T\left(u\right)$ with broadcast power $P_{T}$ defined in Equation (12);
- **Stage 2:** Every node $t\in T\left(u\right)$ can successfully receive the data packet *X*. Then, $T\left(u\right)$ becomes a cooperative transmitting set $\left(|T\left(u\right)|=n\right)$ and cooperatively transmits the packet *X* to node *j* using a joint beamforming vector $w=(w_{1},w_{2},\cdots ,w_{n})$.

**Figure 2:**
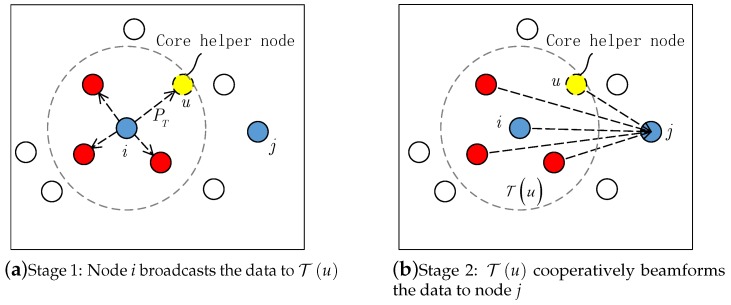
The two-stage cooperative transmission model.

Figure 2 shows the proposed two-stage cooperative transmission model. In Figure 2a, the dotted circle indicates the broadcast range of node *i* and its radius depends on the broadcast power $P_{T}$ as well as the selection of core helper *u* in the first stage. Furthermore, it can be seen from Figure 2b that all the nodes of helper set $T\left(u\right)$ cooperatively beamform the data to node *j* in the second stage.

### 3.2. Two-Stage Link Cost

On the basis of the above two-stage cooperative process, the link cost of the link i→j under core helper *u* consists of two parts:

(14) $$\begin{array}{c} C_{i,u,j}=C_{i,u}+C_{T\left(u\right),j} \end{array}$$

where $C_{i,u}$ and $C_{T\left(u\right),j}$ correspond to the cost of *Stage 1* and *Stage 2*, respectively.

Similar to Equations (7) and (9), the link cost of the first broadcast stage is defined as

(15) $$\begin{array}{c} C_{i,u}={\left(\frac{E_{\mathrm{ini}}}{E_{i}}\right)}^{x}\cdot P_{T}, \end{array}$$

where *x* is a parameter that governs the effect of normalized residual energy.

The packet transmission in the second stage is performed in a general cooperative beamforming manner. Set the cooperative beamforming vector as $w=\{w_{1},w_{2},\cdots ,w_{n}\}$, therefore, in accordance with Equation (15), the link cost of the second stage is defined as

(16) $$\begin{array}{c} C_{T\left(u\right),j}=\min\left\{\sum_{t\in T(u)}{\left(\frac{E_{\mathrm{ini}}}{E_{t}}\right)}^{x}{\left|w_{t}\right|}^{2}\left|\gamma_{j}\geq \gamma_{\min}\right.\right\}, \end{array}$$

which aims at minimizing the total weighted transmission power of $T\left(u\right)$ subject to the condition that node *j* can successfully decode the data. As considered in the Appendix A, by solving this optimization problem, the cooperative beamforming vector w can be designed as

(17) $$\begin{array}{c} {\hat{w}}_{i}=\frac{{\left(E_{i}\right)}^{x}h_{i,j}^{*}}{\sum_{t\in T(u)}{\left(E_{t}\right)}^{x}{\left|h_{t,j}\right|}^{2}}\sqrt{\gamma_{\min}\sigma^{2}},\;i\in T\left(u\right), \end{array}$$

where ${\left(\cdot \right)}^{*}$ stands for the complex conjugate operation. In addition, the minimum value of the total weighted transmission power is given by

(18) $$\begin{array}{c} C_{T(u),j}=\frac{\gamma_{\min}\sigma^{2}}{\sum_{t\in T(u)}{\left(\frac{E_{t}}{E_{\mathrm{ini}}}\right)}^{x}{\left|h_{t,j}\right|}^{2}}. \end{array}$$

The link cost $C_{i,u,j}$ defined in Equation (14) highly depends on the selection of core helper. By selecting the best core helper, the cooperative helpers of the link i→j can be optimized and the number of cooperative nodes can also be determined. The optimized two-stage cost of link i→j under proposed TSC transmission model is then defined as the minimum of $C_{i,u,j}$ over all possible core helpers

(19) $$\begin{array}{c} C_{i,j}^{\mathrm{TSC}}=\min_{u\in U_{i,j}}\left\{C_{i,u,j}\right\}. \end{array}$$

Furthermore, the selection of core helper node is shown in Algorithm 1.

**Algorithm 1** Selection of core helper node 1: Obtain the set of candidate core helpers $U_{i,j}$ for link $P_{i,j}$ according to Equation (11). 2: Traverse $u\in U_{i,j}$, and calculate the related two-stage link cost $C_{i,u,j}$ by Equations (14–16). 3: Determine the core helper with the optimized two-stage link cost $C_{i,j}^{\mathrm{TSC}}$ by Equation (19). 4: The link $P_{i,j}$ owns a core helper $u_{i,j}^{*}⇐\underset{u\in U_{i,j}}{arg\min}\left\{C_{i,u,j}\right\}$.

Algorithm 1 selects the candidate core helper with the optimized two-stage link cost as the core helper $u^{*}$ for the link $P_{i,j}$, and the corresponding helper set $T\left(u^{*}\right)$ can be further determined. In case $u^{*}=i$, node *i* will transmit data directly to *j* with the transmit power given by Equation (17). In this situation, the link cost is completely determined by the second stage and the last term in Equation (14).

It can be seen from Equations (7), (9) and (19) that $C_{i,j}^{\mathrm{MTE}}$ and $C_{i,j}^{\mathrm{FA}}$ are the special cases of $C_{i,j}^{\mathrm{TSC}}$ when $u_{i,j}^{*}=i$, therefore, the link cost of the proposed is smaller or equal than that of MTE and FA. On the other hand, by comparing Equation (18) with (8) and (10), we can see that cost $C_{T(u),j}$ is also smaller than the corresponding cost of PC-*l* and FACR. This is because that the denominators of Equations (18), (8) and (10) will decrease if we limit the transmitter set T to its subset. In our scheme, all nodes which have received the data from *i* and are reachable to *j* can participate in the cooperative beamforming, while in PC-*l* or FACR, only the nodes in the current non-cooperative path are allowed to join.

### 3.3. Two-Stage Cooperative Routing Algorithm

Base on the optimized two-stage link cost defined above, a distributed TSCR algorithm will be illustrated in this subsection.

Suppose that the nodes in the network will periodically broadcast the ’HELLO’ packet to exchange the residual energy and channel information [25] to achieve a periodical topology discovery. Through measuring the received signal strength, each node *i* maintains two *N*-dimensional adjacency vectors, $C_{i}=\left\{C_{i,j}^{\mathrm{TSC}},j\in V\right\}$ records the link cost with other nodes according to Algorithm 1, and $u_{i}=\left\{u_{i,j},j\in V\right\}$ indicates the exact indexes of core helpers corresponding to $C_{i}$.

Suppose that there is a packet *X* to be transmitted from $V_{s}$ to $V_{d}$, denote Q as the set of nodes which have broadcast its own routing request (RREQ) message, and Q is initiated as an empty set. The TSCR algorithm aims at finding a shortest path between $V_{s}$ and $V_{d}$ based on the two-stage link cost in a distributed manner and can be given as follows:

1. Each node i∈V calculates its own adjacency vector $C_{i}$ based on Equation (19) and records the corresponding core helpers $u_{i}$;
2. $V_{s}$ broadcasts its RREQ message where the individual adjacency vector $C_{s}$ is included, $Q=Q\cup V_{s}$;
3. Based on $C_{s}$, find $i=\underset{i\in \{V|Q\}}{\arg\min}\left(C_{s,i}\right)$. If $i\neq V_{d}$, node *i* broadcasts its RREQ message with $C_{i}$, Q=Q∪i;
4. Each node *j* updates $C_{j}$ and records the corresponding route accessing $V_{s}$ ($r_{j,V_{s}}$) based on the following rules: If $C_{s,i}+C_{i,j}<C_{s,j}$, update $C_{s,j}$ as $\left(C_{s,i}+C_{i,j}\right)$ and record $r_{j,V_{s}}$ as j→i→s; otherwise, keep $C_{s,j}$ unchanged;
5. Repeat Step 3 and Step 4 until $i=V_{d}$. Then, $V_{d}$ sends a unicast route reply (RREP) message along with $r_{V_{d},V_{s}}$ to determine the cooperative shortest path.

It can be seen that the shortest path is established based on the optimized two-stage link cost, i.e.,

(20) $$\begin{array}{c} r_{\mathrm{TSCR}}^{*}=\underset{r\in \Omega \left(V_{s},V_{d}\right)}{\arg\min}\sum_{k=1}^{K}C_{r_{k},r_{k+1}}^{\mathrm{TSC}}. \end{array}$$

where *K* denotes the required number of wireless hops.

The source data is then delivered along the routing path $r_{\mathrm{TSCR}}^{*}$. The $k^{\mathrm{th}}$ hop $r_{k}\to r_{k+1}$ performs the two-stage cooperative transmission with $u_{r_{k},r_{k+1}}$ as the core helper. At the first stage, node $r_{k}$ broadcasts data to its neighbors with power $P_{T}$ given by Equation (12). In the second stage, based on Equation (17), the transmitters in helper set $T(u_{r_{k},r_{k+1}})$ jointly beamform the data to node $r_{k+1}$.

## 4. Simulation Results

In this section, the simulations are designed to show the effectiveness of the proposed scheme by comparing its performance with the above-mentioned existing schemes via MATLAB.

We simulate a network with *N* nodes randomly distributed in an $M\times Mm^{2}$ square area. Part of simulation parameters are shown in Table 1 which is similar to the settings in [10,20,29,32]. Unless otherwise specified, all the outputs are based on averaging over 50 randomly generated topologies each with 100 trials. Each trial starts with all nodes initialized with energy $E_{\mathrm{ini}}$ and ends while any one node has depleted its energy. For each trial, communication sessions are continually generated each with random chosen source and destination nodes, and each source has one data packet to be transmitted to the destination. The maximum normalized transmission power is $P_{\max}=400$ units and the initial energy of each node is $E_{\mathrm{ini}}=2000$ units. For the reason of simplicity, we assume that 1 unit transmit power will consume 1 unit energy during one time slot. The end-to-end delay is measured with the total time slots consumed during the communication session. The complex channel gain $h_{i,j}\left(\forall i\;\neq \;j\right)$ is zero-mean complex Gaussian with variance being proportional to $d_{i,j}^{-\alpha }$ where α is the path loss exponent, and $d_{i,j}$ denotes the distance between node *i* and *j*.

In the following simulations, the MTE, PC-3, FA, and FACR schemes are adopted as the baselines for performance comparison. The evaluated performance metrics include the residual energy of the whole network, network lifetime, average end-to-end transmission delay and energy efficiency. We define the dead node as a node whose energy is depleted. The network lifetime is defined as the time when the first dead node appears, and it can be measured with the number of sessions. The average end-to-end transmission delay indicates the average number of time slots consumed when a packet is transmitted from the source to destination. The energy efficiency is the number of packets arriving at destinations divided by the total energy cost during the network lifetime.

In the simulations, the parameter *x* in Equations (15) and (16) can be 0, 1, or 2 and the results are labeled as TSCR-0, TSCR-1 and TSCR-2, respectively.

**Figure 3:**
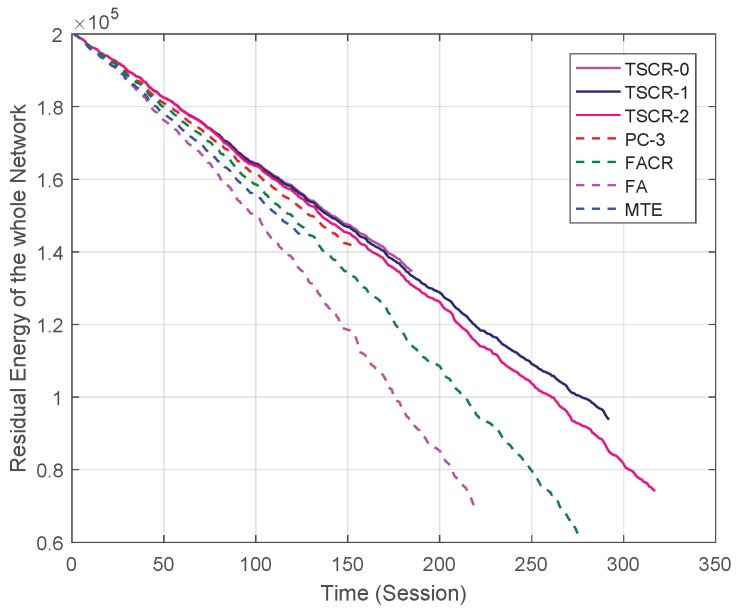
Residual energy of the whole network versus time with different schemes.

The first simulation is to compare the residual energy and lifetime of a fixed network. The results shown in Figure 3 are based on the same fixed network with N=100 nodes and one trial. We can see that TSCR-0 has the best residual energy while TSCR-2 has the longest network lifetime. This is because when x=0, the proposed scheme aims at minimizing the end-to-end energy cost. With a larger *x*, the residual energy of nodes will have larger influence on the link cost, hence the residual energy of nodes can be more balanced and the network lifetime becomes longer. Obviously, minimizing the end-to-end energy cost is not equivalent to maximizing the network lifetime. As mentioned above, the traditional MTE and PC-3 schemes are designed for an excellent end-to-end performance, like the end-to-end energy cost and the end-to-end transmission delay. It can be seen from the comparison between the network residual energy of TSCR-0 scheme and MTE, PC-3 schemes that the proposed cooperative scheme can save more energy. Meanwhile, FA and FACR schemes are designed for prolonging the network lifetime. Compared with FA and FACR schemes, the proposed TSCR-1 and TSCR-2 schemes have achieved a longer network lifetime which reflects a better energy balance in the network.

**Figure 4:**
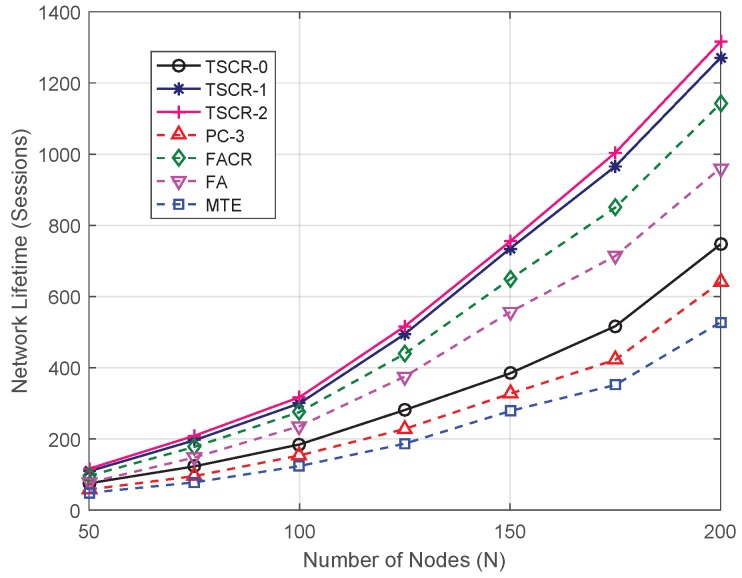
Network lifetime versus different number of nodes.

To further show the effectiveness of proposed TSCR scheme, the network lifetime, average end-to-end transmission delay and energy efficiency are evaluated under different number of nodes *N*. For each point of *N* in Figure 4, the performance is averaged over 50 different network topologies each with 100 trials. It can be seen from the figure that the network lifetime increases with the increase of *N* due to increased total energy in the whole network. Meanwhile, more energy can be saved by cooperative routing because a denser network offers more opportunities for cooperative transmissions. The comparison among TSCR-0, TSCR-1 and TSCR-2 shows that the bigger the *x* is, the longer the network lifetime becomes, which indicates that the increase of *x* can improve the energy balance and prolong the network lifetime. We also observe that when *x* changes from 0 to 1, the network lifetime achieves a big improvement while the change from 1 to 2 brings relatively less gains. Furthermore, on the one hand, both TSCR-1 and TSCR-2 outperform the existing schemes in terms of network lifetime, which proves that proposed TSCR scheme improves the energy balance of network. On the other hand, the higher curve of TSCR-0 over MTE and PC-3 indicates that the proposed TSCR scheme is also effective in saving the energy cost in the end-to-end transmission.

**Figure 5:**
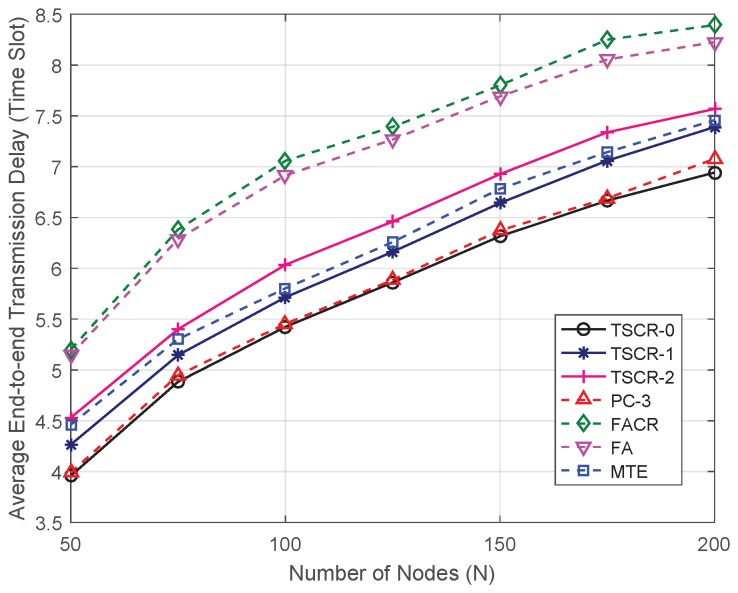
Average end-to-end delay versus different number of nodes.

The average end-to-end transmission delay indicates the end-to-end performance of transmission schemes. The schemes (MTE, PC-3) to minimize the energy cost generally have lower end-to-end transmission delay than the schemes (FA, FACR) aiming at maximizing the network lifetime. Figure 5 shows the average end-to-end transmission delay versus the different number of nodes. It can be seen from the figure that the end-to-end delay increases with the increase of *N*. This is because that lager distance leads to larger signal attenuation, a transmission over multiple short hops requires less power compared with a single transmission over a long distance. Therefore, a denser network will offer more opportunities for energy savings and result in a longer end-to-end transmission delay. Meanwhile, TSCR-0 outperforms all the existing schemes which proves the effectiveness of the proposed TSCR scheme in terms of end-to-end performance. Moreover, the end-to-end transmission delay of TSCR scheme increases as *x* increases, and the end-to-end performance of TSCR-1 and TSCR-2 is lower than PC-3 scheme because the residual energy is considered in the previous two schemes, which leads to a longer network lifetime.

**Figure 6:**
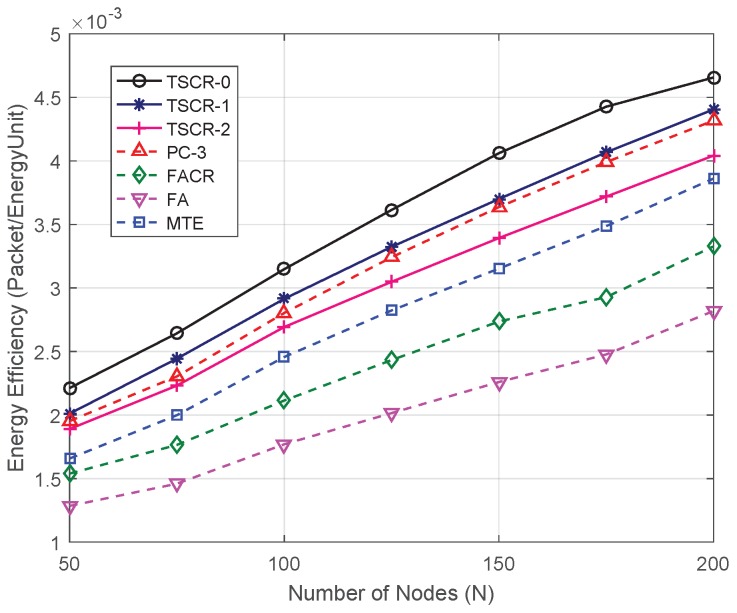
Energy efficiency versus different number of nodes.

Figure 6 shows the energy efficiency of the proposed TSCR scheme compared with the existing schemes. As shown in the figure, the energy efficiency increases with the increase of *N*, this is because the increased nodes can prolong the network lifetime, meanwhile, the increased end-to-end transmission delay indicates the less energy cost per end-to-end transmission. It can be also seen from the figure that the proposed TSCR-0 and TSCR-1 significantly outperform the other schemes in terms of energy efficiency. Moreover, the energy efficiency of proposed TSCR-2 is higher than FA and MTE schemes. The above results prove that the proposed TSCR scheme can save the energy cost of end-to-end transmission and improve the energy balance of network.

In summary, the simulation results indicate that:

1. TSCR-0 has achieved the lowest end-to-end transmission delay and highest energy efficiency. In contrast, TSCR-2 has achieved the longest network lifetime; Meanwhile, the network lifetime of TSCR-0 is better than that of MTE and PC-3, and the end-to-end transmission performance of TSCR-1 and TSCR-2 is better than that of FA and FACR, which shows that TSCR scheme can improve the end-to-end performance and network lifetime simultaneously.
2. Compared with MTE and PC-3, TSCR-2 has higher end-to-end transmission delay and lower energy efficiency, while the network lifetime of TSCR-0 is lower than the FA and FACR. These prove the trade-off between end-to-end energy cost and network balance;
3. Since TSCR-1 has better energy efficiency and longer network lifetime than all the existing schemes, it can be regarded as a good trade-off between end-to-end performance and network energy balance.
4. With different setting of *x*, the proposed TSCR scheme can meet different performance requirements.

## 5. Conclusions

In this paper, we focused on the optimal cooperative routing path from a source node to a destination node through multi-hop wireless transmission. A TSC transmission model was proposed and the cooperative link cost was formulated. Based on the proposed two-stage link cost, a distributed TSCR scheme was proposed to achieve a high energy-efficient packet transmission and prolong the network lifetime. Simulation results have verified the effectiveness of the proposed TSCR scheme on the energy savings and energy balance. Compared with the existing schemes, the proposed TSCR can simultaneously prolong the network lifetime, shorten the end-to-end transmission delay and improve the energy efficiency. In addition, the proposed TSCR scheme can be adjusted to accommodate the different performance goals through the parameter *x*.

It has been assumed in this paper that there is only one active communication session in the whole network. The future work will extend the proposed TSCR scheme to multiple-source multi-destination scenario as studied in [27,28]. To this end, the interference between different data flows needs to be carefully addressed.

## Figures and Tables

**Table 1:** Parameters for simulation.

Parameter	Value	Parameter	Value
*M*	100	$E_{\mathrm{ini}}$	2000
$P_{\max}$	400	α	2
$\gamma_{\min}$	1	$\sigma^{2}$	1

## Appendix A. Cooperative Beamforming Model

Our cooperative transmission model in the second stage assumes *n* transmitters $T=\left\{t_{1},\cdots ,t_{n}\right\}$ and a single receiver *j*. In this sense, the model is similar to the MISO case. The following analysis has been inspired by [19].

The transmitters in T beamform the packet to the receiver and $w=\{w_{1},w_{n},\cdots ,w_{n}\}$ denotes the cooperative beamforming vector. The link cost of the cooperative transmission is designed as the total transmit power weighted by the normalized residual energy: $C_{T,j}=\sum_{i=1}^{n}{\left(\frac{E_{\mathrm{ini}}}{E_{i}}\right)}^{x}{\left|w_{i}\right|}^{2}$. The problem of cooperative link cost minimization subject to that node *j* can successfully receive the packet can be expressed as

(A1) $$\begin{array}{c} \begin{array}{c} \min\;C_{T,j}\\ s.t.\;\frac{{\left|\sum_{i=1}^{n}h_{i,j}w_{i}\right|}^{2}}{\sigma^{2}}\geq \gamma_{\min}. \end{array}. \end{array}$$

Using the Lagrange multiplier method we have

(A2) $$\begin{array}{c} L\left(w_{1},\cdots ,w_{n},\lambda \right)=\sum_{i=1}^{n}{\left(\frac{E_{\mathrm{ini}}}{E_{i}}\right)}^{x}{\left|w_{i}\right|}^{2}-\lambda \left(\frac{{\left|\sum_{i=1}^{n}h_{i,j}w_{i}\right|}^{2}}{\sigma^{2}}-\gamma_{\min}\right). \end{array}$$

Take the partial derivatives for $w_{i},i=\left\{1,\cdots ,n\right\}$ and λ, respectively, the following n+1 equations can be obtained.

(A3) $$\begin{array}{c} \frac{\partial L}{\partial w_{i}}=2{\left(\frac{E_{\mathrm{ini}}}{E_{i}}\right)}^{x}w_{i}-2\frac{\lambda h_{i,j}\left|\sum_{i=1}^{n}h_{i,j}w_{i}\right|}{\sigma^{2}}=0,\;i=\left\{1,\cdots ,n\right\}, \end{array}$$

and

(A4) $$\begin{array}{c} \frac{\partial L}{\partial \lambda }=\frac{{\left|\sum_{i=1}^{n}h_{i,j}w_{i}\right|}^{2}}{\sigma^{2}}-\gamma_{\min}=0. \end{array}$$

Combine Equations (A3) and (A4), the solutions of λ can be solved as

(A5) $$\begin{array}{c} \hat{\lambda }=\frac{\sigma^{2}}{\sum_{i=1}^{n}{\left|h_{i,j}\right|}^{2}{\left(\frac{E_{i}}{E_{\mathrm{ini}}}\right)}^{x}}, \end{array}$$

Substitute $\hat{\lambda }$ into Equation (A4), $w_{i}$ can be solved

(A6) $$\begin{array}{c} {\hat{w}}_{i}=\frac{{\left(E_{i}\right)}^{x}h_{i,j}^{*}\sqrt{\sigma^{2}\cdot \gamma_{\min}}}{\sum_{t=1}^{n}{\left(E_{t}\right)}^{x}{\left|h_{t,j}\right|}^{2}},i=\left\{1,\cdots ,n\right\}. \end{array}$$

where ${\left(\cdot \right)}^{*}$ stands for the complex conjugate operation.

## References

[1] Andrews J.G., Buzzi S., Choi W., Hanly S.V., Lozano A., Soong A.C.K., Zhang J. (2014). What Will 5G Be?. IEEE J. Sel. Areas Commun..

[2] Akkaya K., Younis M. (2005). A survey on Routing Protocols for Wireless Sensor Network. Ad Hoc Netw. J..

[3] Taneja S., Kush A. (2010). A Survey of Routing Protocols in Mobile Ad Hoc Networks. Int. J. Innov. Manag. Technol..

[4] Raymond J.W., Olwal T.O., Kurien A.M. (2018). Cooperative Communications in Machine to Machine (M2M): Solutions, Challenges and Future Work. IEEE Access.

[5] Mansourkiaie F., Ahmed M.H. (2015). Cooperative Routing in Wireless Networks: A Comprehensive Survey. IEEE Commun. Surv. Tutor..

[6] Hunter T.E., Hedayat A. (2004). Cooperative communication in wireless networks. IEEE Commun. Mag..

[7] Taghizadeh O., Radhakrishnan V., Alirezaei G., Mathar R. Partial Distributed Beamforming Design in Passive Radar Sensor Networks. Proceedings of the 2016 IEEE International Conference on Wireless for Space and Extreme Environments (WiSEE).

[8] Yan J., Zhou M., Ding Z. (2016). Recent Advances in Energy-Efficient Routing Protocols for Wireless Sensor Networks: A Review. IEEE Access.

[9] Toh C.K. (2001). Maximum battery life routing to support ubiquitous mobile computing in wireless ad hoc networks. IEEE Commun. Mag..

[10] Khandani A.E., Abounadi J., Modiano E., Zheng L. (2007). Cooperative Routing in Static Wireless Networks. IEEE Trans. Commun..

[11] Elhawary M., Haas Z.J. (2011). Energy-Efficient Protocol for Cooperative Networks. IEEE Trans. Netw..

[12] Li F., Wu K., Lippman A. Energy-Efficient Cooperative Routing in Multi-hop Wireless Ad Hoc Networks. Proceedings of the 2006 IEEE International Performance Computing and Communications Conference.

[13] Shi L., Fapojuwo A.O. (2012). Cross-layer optimization with cooperative communication for minimum power cost in packet error rate constrained wireless sensor networks. Ad Hoc Netw..

[14] Shi J., Calveras A., Cheng Y., Liu K. (2013). A novel power efficient location-based cooperative routing with transmission power-upper-limit for wireless sensor networks. Sensors.

[15] Dehghan M., Ghaderi M., Goeckel D. (2011). Minimum-Energy Cooperative Routing in Wireless Networks with Channel Variations. IEEE Trans. Wirel. Commun..

[16] Cherkassky B.V., Goldberg A.V., Radzik T. (1996). Shortest Paths Algorithms: Theory and Experimental Evaluation. Math. Program..

[17] Yetgin H., Cheung K.T.K., El-Hajjar M., Hanzo L.H. (2017). A Survey of Network Lifetime Maximization Techniques in Wireless Sensor Networks. IEEE Commun. Surv. Tutor..

[18] Chang J.H., Tassiulas L. (2004). Maximum Lifetime Routing in Wireless Sensor Networks. IEEE/ACM Trans. Netw..

[19] Pandana C., Siriwongpairat W.P., Himsoon T., Liu K.J.R. Distributed Cooperative Routing Algorithms for Maximizing Network Lifetime. Proceedings of the 2006 IEEE Wireless Communications and Networking Conference (IEEE WCNC 2006).

[20] Chen S., Li Y., Huang M., Zhu Y., Wang Y. (2013). Energy-balanced cooperative routing in multihop wireless networks. Wirel. Netw..

[21] Taghizadeh O., Aorezaei G., Mathar R. Lifetime and Power Consumption Optimization for Distributed Passive Radar Systems. Proceedings of the 2016 IEEE International Conference on Wireless for Space and Extreme Environments (WiSEE).

[22] Zhai C., Liu J., Zheng L., Xu H., Chen H. (2012). Maximise lifetime of wireless sensor networks via a distributed cooperative routing algorithm. Trans. Emerg. Telecommun. Technol..

[23] Zhang J., Zhang D., Xie K., Qiao H., He S. (2017). A VMIMO-Based Cooperative Routing Algorithm for Maximizing Network Lifetime. China Commun..

[24] Goldsmith A. (2005). Wireless Communiations.

[25] Ibrahim A., Han Z., Liu K. (2008). Distributed energy-efficient cooperative routing in wireless networks. IEEE Trans. Wirel. Commun..

[26] Mudumbai R., Brown D.R., Madhow U., Poor H.V. (2009). Distributed Transmit Beamforming: Challenges and Recent Progress. IEEE Commun. Mag..

[27] Xie K., Wang X., Liu X., Wen J., Cao J. (2016). Interference-Aware Cooperative Communication in Multi-Radio Multi-Channel Wireless Networks. IEEE Trans. Comput..

[28] Li P., Guo S., Hu J. (2015). Energy-Efficient Cooperative Communications for Multimedia Applications in Multi-Channel Wireless Networks. IEEE Trans. Comput..

[29] Dehghan M., Ghaderi M., Goeckel D.L. On the performance of Cooperative Routing in Wireless Networks. Proceedings of the 2010 IEEE Conference on Computer Communications Workshops (IEEE INFOCOM 2010).

[30] Taghizadeh O., Sirvi P., Narasimha S., Calvo J.A.L., Mathar R. Minimum-cost wireless backhaul network planning with Full-Duplex links. Proceedings of the 2018 IEEE Wireless Communications and Networking Conference (WCNC).

[31] Taghizadeh O., Sirvi P., Narasimha S., Calvo J.A.L., Mathar R. (2018). Environment-Aware Minimum-Cost Wireless Backhaul Network Planning with Full-Duplex Links. arXiv.

[32] Tirronen T., Larmo A., Sache J., Lindoff B., Wiberg N. (2013). Machine-to-machine communication with long-term evolution with reduced device energy consumption. Trans. Emerg. Telecommun. Technol..

